# Novel biopesticide based on a spider venom peptide shows no adverse effects on honeybees

**DOI:** 10.1098/rspb.2014.0619

**Published:** 2014-07-22

**Authors:** Erich Y. T. Nakasu, Sally M. Williamson, Martin G. Edwards, Elaine C. Fitches, John A. Gatehouse, Geraldine A. Wright, Angharad M. R. Gatehouse

**Affiliations:** 1 School of Biology, Newcastle Institute for Research and Sustainability, Newcastle University, Newcastle upon Tyne NE1 7RU, UK; 2 Institute of Neuroscience, Newcastle University, Newcastle upon Tyne NE1 7RU, UK; 3 Capes Foundation, Ministry of Education of Brazil, Caixa Postal 250, Brasília 70040-020, Brazil; 4 The Food and Environment Research Agency, Sand Hutton, York YO41 1LZ, UK; 5 School of Biological and Biomedical Sciences, Durham University, South Road, Durham DH1 3LE, UK

**Keywords:** insecticidal fusion proteins, biopesticide, honeybees, ω-hexatoxin-Hv1a, snowdrop lectin (*Galanthus nivalis* agglutinin), pollinator decline

## Abstract

Evidence is accumulating that commonly used pesticides are linked to decline of pollinator populations; adverse effects of three neonicotinoids on bees have led to bans on their use across the European Union. Developing insecticides that pose negligible risks to beneficial organisms such as honeybees is desirable and timely. One strategy is to use recombinant fusion proteins containing neuroactive peptides/proteins linked to a ‘carrier’ protein that confers oral toxicity. Hv1a/GNA (*Galanthus nivalis* agglutinin), containing an insect-specific spider venom calcium channel blocker (ω-hexatoxin-Hv1a) linked to snowdrop lectin (GNA) as a ‘carrier’, is an effective oral biopesticide towards various insect pests. Effects of Hv1a/GNA towards a non-target species, *Apis mellifera*, were assessed through a thorough early-tier risk assessment. Following feeding, honeybees internalized Hv1a/GNA, which reached the brain within 1 h after exposure. However, survival was only slightly affected by ingestion (LD_50_ > 100 µg bee^−1^) or injection of fusion protein. Bees fed acute (100 µg bee^−1^) or chronic (0.35 mg ml^−1^) doses of Hv1a/GNA and trained in an olfactory learning task had similar rates of learning and memory to no-pesticide controls. Larvae were unaffected, being able to degrade Hv1a/GNA. These tests suggest that Hv1a/GNA is unlikely to cause detrimental effects on honeybees, indicating that atracotoxins targeting calcium channels are potential alternatives to conventional pesticides.

## 1. Introduction

Pest control is an essential component of food security and agricultural productivity, as herbivorous pests, weeds and pathogens can cause significant losses in staple food crops unless control measures are in place [1]. Since the 1940s, crop protection from insect pests has been reliant on synthetic chemical insecticides such as DDT and organophosphates [2]; these chemicals improved yields, but with a cost of negative consequences for non-target organisms, including humans [3]. To overcome this, industrial producers have designed pesticides such as synthetic pyrethroids, neonicotinoids and growth regulators with greater specificity for targeted pests that are now used worldwide [4]. Neonicotinoids are general agonists of insect nicotinic acetylcholine receptors, but bind only weakly to homologous receptors in higher animals [5]. Their efficacy and low mammalian toxicity have led to their widescale adoption, and they currently make up 24% of the world insecticide market [6]. However, several reports of adverse effects of neonicotinoids on beneficial pollinating insects [7,8] have recently resulted in a controversial ban of the use of three neonicotinoid pesticides (clothianidin, thiamenthoxam and imidacloprid) by the European Commission. Insect pollination is an important ecosystem service, but it is also essential for fruit set in many crop species, contributing to 35% of global food production in approximately 70% of crops [9]. Sublethal exposure to nectar-relevant doses of neonicotinoids impairs the function of Kenyon cells in the honeybee's mushroom bodies [10] and reduces olfactory learning and memory [7,11] and homing ability [12]. In bumblebees, field-relevant, sublethal doses of these pesticides reduce foraging success and cause failure of bee colonies [13]. While neonicotinoids and other chemical pesticides clearly have negative impacts on pollinating bee species [13,14], banning them without more appropriate alternatives could have significant consequences for food production or biodiversity, if less specific pesticides are used to replace them.

Potential alternatives to neonicotinoids and other chemical pesticides include the development and use of biopesticides: biological agents or bioactive compounds that often have high specificity for target pest species [15]. Examples of currently used biopesticides include entomopathogenic fungi [16], and toxins derived from the entomopathogenic bacterium *Bacillus thuringiensis* [17]. Biopesticide candidates such as the venom of predatory arthropods that target the voltage-gated calcium ion channels (CaV) are very potent and selective [18]. Since CaV channels are not highly conserved in insects, this makes them attractive alternatives and represents a novel mode of action to conventional pesticides.

Fusion protein technology, in which insecticidal peptides are linked to a plant lectin ‘carrier’ protein, has been developed to allow proteins such as spider venom toxins to act as orally delivered biopesticides. For example, ω-hexatoxin-Hv1a (Hv1a; also referred to elsewhere as ω-atracotoxin-Hv1a or ω-ACTX-Hv1a) from the Australian funnel web spider *Hadronyche versuta* acts on CaV channels in the insect central nervous system (CNS), causing paralysis [19]. This toxin is lethal to many insect species when injected, but does not affect mammals [20]. When delivered orally it is essentially non-toxic to insects, as it is unable to reach its site of action in the CNS. Fusion of this insecticidal molecule to the carrier protein snowdrop lectin (*Galanthus nivalis* agglutinin, GNA), allows Hv1a to traverse the insect gut epithelium and access its sites of action, producing an orally active insecticidal protein [21]. The Hv1a/GNA fusion protein has oral insecticidal activity against insects from a range of orders, including Lepidoptera, Coleoptera, Diptera and Hemiptera.

Fusion protein biopesticides have the potential to improve pest management strategies, but they have not yet been tested on important insect pollinators such as bee species. In Europe, laboratory-risk assessments of pesticides on bees currently include determination of acute contact and oral toxicity on adult honeybees, following the guidelines from the European and Mediterranean Plant Protection Organization 170 [22] and Organisation for Economic Co-operation and Development (OECD) 213 and 214 [23,24]. Despite conforming to these criteria for assessing pesticide toxicity to bees, pesticides can also exert a range of effects on pollinator behaviour at sublethal and field-realistic concentrations that are not detectable by current guidelines [25,26]. For example, subtle aspects of bee behaviour important for foraging and survival, such as learning and memory, can be impaired after prolonged exposure to pesticides [7,8]. It is therefore sensible to assume that more rigorous testing of pesticide toxicity to pollinating insects should be implemented alongside the development of new biopesticide products, to identify risks prior to their implementation in the field and to reduce environmental impact.

Here, we report the testing of the insecticidal fusion protein Hv1a/GNA for toxicity to honeybees including the recommended acute toxicity tests from the OECD guidelines and in a test of cognitive function under both acute and long-term exposure. We also address the issues involved in testing pesticides on pollinators, suggesting that additional toxicity tests, such as a chronic toxicity assay, and an evaluation of any potential effects which pesticides may have on honeybee behaviour should be adopted to assess critical factors for bee viability and their role as pollinators.

## 2. Material and methods

### (a) Honeybees

Honeybee colonies (*Apis mellifera mellifera*) were originally obtained from the National Bee Unit, York, UK, and were then maintained at Newcastle University. During the summer months (April–October 2012), bees were kept outdoors and allowed to fly and forage freely. During the winter months (November 2012–March 2013), bees were maintained indoors, but were still allowed to fly freely via a plastic pipe connecting the hive entrance to the outdoors.

### (b) Pesticides and toxins

Recombinant GNA, and the fusion protein Hv1a/GNA were produced in the yeast expression system *Pichia pastoris* as previously described [21,27]. The pesticide thiamethoxam (TMX) (Sigma Aldrich, 99% purity) and the CaV channel blocker benidipine HCl (Tocris Bioscience) were dissolved directly in 1 M sucrose solution for oral administration to adult forager bees. Acetamiprid (Ace) (Scotts) was obtained as a liquid formulation (0.5% Ace, 1–5% ethanol, less than 1% of aqueous dipropylene glycol solution of approx. 20% 1,2-benzisothiazolin-3-one, 5–10% glycerol).

### (c) Toxicity studies

#### (i) Acute toxicity tests of Hv1a/GNA

Acute toxicity was assessed by injection, and by oral and contact bioassays, using adult forager honeybees. Bees were collected from outside the hive in small plastic vials and then cold anaesthetized to allow manipulation or transference to containers.

After all acute toxin administration regimes (see below), bees were kept in 650 ml plastic storage containers fitted with 2 ml microcentrifuge tubes that had four holes drilled in for bee access. Bees were kept at 25°C in the dark and allowed to feed ad libitum on 50% w/v sucrose solution. Mortality was recorded at 4, 24 and 48 h after exposure to the test compound.

Acute oral and contact toxicity assays were performed according to the OECD guidelines [23,24]. For contact toxicity assays, bees were cold anaesthetized and individually treated by topical application of phosphate-buffered saline—Tween (PBST; 137 mM NaCl, 2.7 mM KCl, 10 mM Na_2_HPO_4_.2H_2_O, 3 mM KH_2_PO_4_, pH 7.4, containing 0.05% Tween-20; negative control), GNA in PBST (20 μg bee^−1^), Hv1a/GNA in PBST (20 μg bee^−1^) or Ace as the positive control (4, 8.09 or 16.18 μg bee^−1^, in PBST), directly applied to the thorax using a micropipette. After application, insects were separated into storage boxes as described above. Ten bees were used per treatment, and each treatment replicated seven times.

For the acute oral toxicity assays, insects were starved for 2 h prior to testing, in order to encourage active feeding during the assay. Bees were collected, cold anaesthetized and placed inside the storage containers, in replicates of 10 individuals per container. After starvation bees were fed via a feeder with either 200 µl of sucrose (50% w/v) solution (negative control), or sucrose solution containing GNA (control; 100 µg bee^−1^), Hv1a/GNA (100 µg bee^−1^), or Ace (positive control; 7.26, 14.52 or 29 µg bee^−1^). Insects were allowed to feed, without restraint, on the treatments for up to 4 h, after which these feeders were removed and replaced with sucrose solution (50% w/v) feeders to allow feeding ad libitum. Six replicates of 10 bees were used for the negative control, GNA and Hv1a/GNA treatments, whereas four replicates of 10 bees were used for each concentration of the positive control.

Effects of the recombinant proteins were also evaluated by an injection bioassay. Adult honeybees (30 per treatment) were cold anaesthetized and injected into the thorax with either (i) 5 µl of phosphate-buffered saline (PBS; as described above); (ii) 5 µl of a 4 µg µl^−1^ GNA solution in PBS buffer (20 µg of GNA bee^−1^) or (ii) 5 µl of a 4 µg µl^−1^ Hv1a/GNA solution in PBS buffer (20 µg of Hv1a/GNA bee^−1^) using a Hamilton syringe (Model 25F, needle gauge 25). After injection, bees were divided into groups of 10 inside the storage containers.

#### (ii) Chronic toxicity tests of Hv1a/GNA

Bees were collected, anaesthetized, then transferred to storage containers with feeding tubes as described above. Bees were allowed to feed ad libitum for 7 days on one of three treatment solutions: (i) 1 M sucrose, (ii) 350 µg ml^−1^ Hv1a/GNA in 1 M sucrose, or (iii) 10 ng ml^−1^ TMX in 1 M sucrose. Bees were maintained in an incubator at 34°C for the duration of the treatment period, and mortality was recorded daily. Sample size was 40 bees per treatment group.

#### (iii) Testing of Hv1a/GNA for acute toxicity towards honeybee larvae

Standard operating procedures established for the *in vitro* testing of pesticides were used to test for acute toxicity of Hv1a/GNA towards honeybee larvae [28]. A single oral dose of 100 μg larva^−1^ of Hv1a/GNA was administered to 4 day-old larvae individually maintained in microtitre plate wells. Plates were incubated under controlled environmental conditions at 34°C in the dark, 60% relative humidity. A total of 30 larvae were treated alongside a control treatment, in which larvae were fed on a diet with no added protein. Fifteen larvae were sacrificed at 24 and 92 h after exposure to the fusion protein to obtain haemolymph, whole larval and diet samples for western blot analysis to assess the stability of the fusion protein. Haemolymph (at least 5 µl per insect) was obtained by piercing pre-chilled larvae with a fine needle and collecting into pre-chilled phenylthiocarbamide-phenol oxidase inhibitor to prevent melanization. The survival of the remaining 15 larvae was monitored for 4 days subsequent to the single acute Hv1a/GNA dose.

### (d) Behavioural studies

#### (i) Acute Hv1a/GNA exposure for learning and memory experiments

Forager bees were collected from outside the hive in small plastic vials, cold anaesthetized and restrained in harnesses [29]. The bees were fed 20 µl of 1 M sucrose solution, then left overnight to become sufficiently hungry and motivated to perform the olfactory learning task. One hour prior to the learning task, each bee was fed 5 µl of treatment solution. The treatment groups were: (i) a control group fed 5 µl of 1 M sucrose; (ii) 100 µg of Hv1a/GNA in 5 µl of 1 M sucrose; (iii) 100 µg of GNA in 5 µl of 1 M sucrose; and (iv) 500 ng of benidipine HCl in 5 µl of 1 M sucrose. The experiment was repeated with three cohorts, and the total sample size of trained bees was greater than or equal to 20 bees per treatment group.

#### (ii) Long-term Hv1a/GNA exposure for learning and memory experiments

Foraging worker bees were collected and cold anaesthetized. Ten bees were transferred to each feeding box (16.5 × 11 × 6.5 cm) fitted with 2 ml microcentrifuge tubes with evenly spaced holes for feeding the solutions. Bees were allowed to feed ad libitum for 7 days on one of three treatment solutions: (i) 1 M sucrose, (ii) 350 µg ml^−1^ Hv1a/GNA in 1 M sucrose, or (iii) 10 ng ml^−1^ TMX (i.e. 10 ppb or 34 nM) in 1 M sucrose. Bees were maintained in an incubator at 34°C for the duration of the treatment period, and mortality was recorded daily. After this, the bees were cold anaesthetized and restrained in harnesses, fed 20 µl of treatment solution and left overnight to become sufficiently motivated to perform the olfactory learning task. The survival analysis was repeated four times (*n* = 40 per treatment group). A subset of bees was selected from these cohorts for the olfactory conditioning assay.

#### (iii) Learning and memory experiments

An olfactory conditioning protocol based on the proboscis extension reflex (PER) was performed [29]. The conditioned stimulus (CS; 1-hexanol) and unconditioned stimulus (0.2 µl of 1 M sucrose solution) were presented for six training trials, with a 10 min inter-trial interval. PER response to the CS was recorded. Two unreinforced recall tests (the CS and a novel odour) were administered at 10 min after conditioning and again at 24 h. The order of presentation of these two test stimuli was pseudorandomized across subjects.

### (e) Detection of Hv1a/GNA in honeybee tissues by western blotting

To test internalization of recombinant proteins, tissue samples were collected from bees following 24 h feeding on either GNA or Hv1a/GNA, as described above, using a modified version of the method described by Mayack & Naug [30]. For haemolymph from adults, insects were killed at −20°C and immediately wrapped with Parafilm. The distal end of one of the antennae was cut and insects were placed individually in microcentrifuge tubes. Tubes were spun for 30 s at 5000*g* and haemolymph collected and kept at −80°C until use. Haemolymph was collected from larvae previously exposed to the recombinant proteins after either 24 h (5 days-old larvae) or 92 h (8 days-old larvae), as detailed above. For brain samples from adults, insects were cold anaesthetized, restrained in harnesses and fed with 20 μl of 1 M sucrose solution (negative control) or 100 μg Hv1a/GNA in 20 μl of 1 M sucrose solution. After 24 h, honeybees were freeze-killed and the brains removed. Six brains from each treatment were pooled and macerated in sodium dodecyl sulfate (SDS) sample buffer (100 mM Tris–HCl, pH 6.8, 4% SDS, 9% glycerol, 2% 2-mercaptoethanol, 0.001% bromophenol blue). Proteins from individual samples were separated in 15% SDS-polyacrylamide gel electrophoresis (SDS-PAGE), transferred to nitrocellulose membranes and screened for the presence of GNA or Hv1a/GNA by SDS-PAGE followed by western blotting using anti-GNA antibodies [21].

### (f) Statistical analysis

Log-rank Kaplan–Meier (K–M) survival analyses with pairwise comparisons over strata were carried out using SPSS v. 19.0. The median lethal dose (LD_50_) with 95% confidence intervals (CIs) for positive controls on acute oral and contact bioassays were estimated by plotting log dose versus probit of corrected mortalities [31–33]. PER response during the learning and memory tests was scored as a binary response, and data were analysed in SPSS using a binary logistic regression (lreg). Data from the first training trial were excluded from the analysis to facilitate model fit. Pairwise comparisons between different treatments, time points and odours were performed using least-squares *post hoc* comparisons (lsc). PER data represent the mean probability of responding with a Wald χ^2^ 95% CI.

## 3. Results

### (a) Testing the acute and chronic toxicity of Hv1a/GNA to honeybees

**Figure 1.**
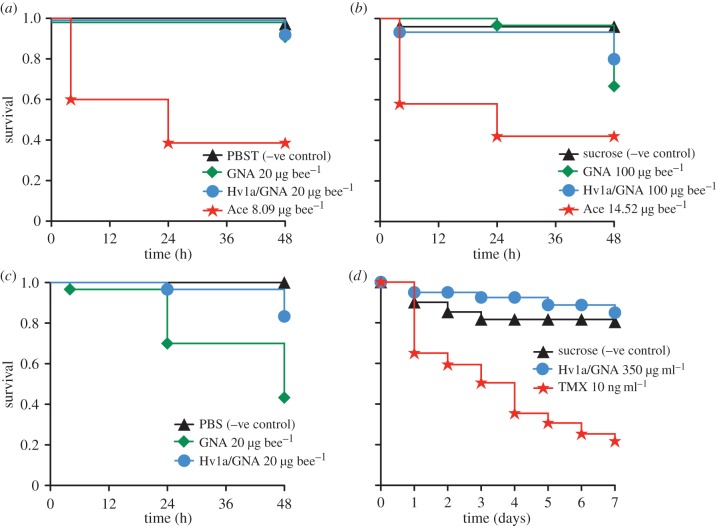
Survival analyses indicate Hv1a/GNA poses no substantial toxicity towards adult honeybees. (*a*) Acute contact toxicity assay of GNA and Hv1a/GNA with honeybees (20 µg of test protein per bee; *n* = 70 bees per treatment). Survival curve for the positive control acetamiprid (Ace) (8.09 µg bee^−1^) is shown. (*b*) Acute oral toxicity bioassays of GNA (*n* = 60) and Hv1a/GNA (*n* = 60) with honeybees (100 µg of test protein per bee). Survival curve for positive control Ace (14.52 µg bee^−1^, *n* = 40) is shown. (*c*) Effects of GNA and Hv1a/GNA on survival of honeybees following injection (20 μg of test protein per bee; *n* = 30 bees per treatment). (*d*) Honeybee survival was unaffected by chronic consumption of 21.7 µg bee^−1^ day^−1^ dose of Hv1a/GNA, but a 0.727 ng bee^−1^ day^−1^ dose of thiamethoxam (TMX) increased mortality (*n* = 40 bees per treatment). Dose–response curves for both acute contact and acute oral bee toxicity assays for all Ace concentrations are presented in the electronic supplementary material, figure S1*a*,*b*, respectively. (Online version in colour.)

In order to assess the potential toxicity of Hv1a/GNA to pollinators, bioassays were carried out to measure the survival of honeybees after exposure to the fusion protein (figure 1). The Hv1a/GNA treatment regimens included acute contact and oral exposure, acute injection, and a chronic 7-days oral exposure; the neonicotinoids Ace and TMX, were used to compare mortality caused by a neonicotinoid to that of the fusion protein.

In the acute contact toxicity assays, the positive control Ace induced bee mortality when compared to the negative control (PBST), GNA control or Hv1a/GNA treatments (figure 1*a*; K–M, PBST versus Ace, 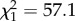, *p* < 0.001; Hv1a/GNA versus Ace, 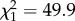, *p* < 0.001; GNA versus Ace, 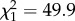, *p* < 0.001), with an estimated LD_50_ of 6.78 ± 0.58 μg bee^−1^, thus within the limits reported in the literature [34]. When compared to the negative control, neither Hv1a/GNA nor GNA increased mortality after contact exposure (K–M, Hv1a/GNA, 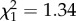, *p* = 0.246; GNA, 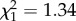, *p* = 0.246) when applied at 20 µg bee^−1^. It is unlikely that the fusion protein or the GNA are able to cross the insect cuticle, and thus a lack of toxicity in this assay is expected.

In the acute oral treatments with the compounds, bees fed the neonicotinoid, Ace, were the least likely to survive of all treatments (figure 1*b*; K–M, sucrose versus Ace, 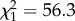, *p* < 0.001). The estimated LD_50_ for this compound was 8.95 ± 0.23 µg bee^−1^, which is comparable to those reported for formulated products [35]. Survival of honeybees fed on Hv1a/GNA or GNA at the maximum recommended dose for oral toxicity assays (100 μg bee^−1^) was reduced by 22% for the fusion protein (K–M, sucrose versus Hv1a/GNA, 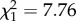, *p* = 0.005) and 34% for the GNA (K–M, sucrose versus GNA, 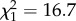, *p* < 0.001). Survival of the bees fed either Hv1a/GNA or GNA was greater than those fed acetamiprid (K–M, Hv1a/GNA versus Ace, 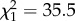, *p* < 0.001; GNA versus Ace, 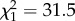, *p* < 0.001). We can therefore conclude that Hv1a/GNA and GNA are of relatively low toxicity to honeybees as the oral LD_50_ > 100 μg/bee. An acute toxicity assay was also performed on larval honeybees: no mortality was observed for either control or Hv1a/GNA treatments, with 100% survival recorded 4 days post-treatment.

In order to exclude the possibility that low toxicity of Hv1a/GNA was owing to inefficient transport of the Hv1a/GNA from the gut to the haemolymph, toxicity of Hv1a/GNA and GNA by injection was assessed to represent a ‘worst case scenario’. In this test, injections were of 20 μg protein bee^−1^. The mortality over 48 h was greatest for those injected with GNA (57% mortality; figure 1*c*; K–M, PBS versus GNA, 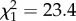, *p* < 0.001; GNA versus Hv1a/GNA, 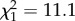, *p* = 0.001). While bees injected with Hv1a/GNA also had significantly greater mortality than the PBS control (K–M, PBS versus Hv1a/GNA, 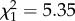, *p* = 0.021), mortality levels were relatively low (<17%). These low levels were similar to the acute oral treatment, confirming that only a very high dose of this compound could produce measurable mortality in honeybees. Most of this mortality occurred between the 24 and 48 h time points.

Previously, the Hv1a/GNA fusion protein has been shown to be an effective insecticide when used as a foliar spray; the protein is stable over timescales more than two weeks under these conditions and provides continuing protection without the need for re-spraying (E. C. Fitches 2013, unpublished data). The toxicity of chronic consumption of Hv1a/GNA at the effective concentration when delivered as a spray, 350 ppm (0.35 mg ml^−1^), by adult forager honeybees was also investigated, and compared directly to the chronic toxic effects of the neonicotinoid, TMX, at the concentrations reported in the nectar and pollen of treated crops [36,37]. Each bee consumed on average 63.8 ± 0.003 µl of the control solution, 62.1 ± 0.002 µl of the Hv1a/GNA solution and 72.7 ± 0.004 µl of the TMX solution per day. Based on the average volume of solution consumed per day, the estimated dose of the Hv1a/GNA solution for each bee was 21.7 µg bee^−1^ day^−1^, and the estimated dose of the thiamethoxam for each bee was 0.727 ng bee^−1^ day^−1^. After 7 days of treatment, TMX treatment significantly increased mortality compared to the other groups (figure 1*d*; K–M, sucrose versus TMX, 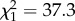, *p* < 0.001). In contrast to this, there was no difference in survival between the control group and the Hv1a/GNA treatment group (K–M, sucrose versus Hv1a/GNA, 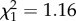, *p* = 0.282), again confirming low toxicity of Hv1a/GNA to honeybees.

### (b) Testing the effects of Hv1a/GNA on honeybee learning and memory

**Figure 2.**
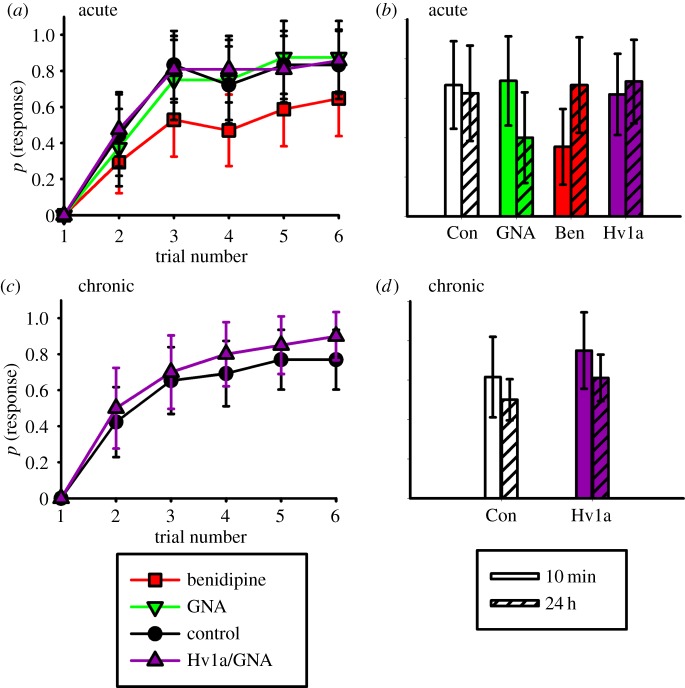
Hv1a/GNA consumption does not affect honeybee learning and memory. (*a*) The rate of learning is reduced in the positive control (the calcium channel blocker, benidipine HCl; Ben), whereas acute exposure to Hv1a/GNA (Hv1a), or GNA, does not significantly influence olfactory learning relative to the control (Con). *N*_control_ = 20, *N*_GNA_ = 20, *N*_Ben_ = 23, *N*_Hv1a/GNA_ = 23. (*b*) Short term memory (STM) was impaired for the Ben group, but not for the other treatments (lsc comparisons against the control: GNA, *p* = 0.740, Ben, *p* = 0.025, Hv1a/GNA, *p* = 0.661). (*c*) The rate of learning was not significantly different for bees fed Hv1a/GNA for 7 days. *N*_control_ = 26, *N*_Hv1a/GNA_ = 20. (*d*) STM (10 min) and long term memory (24 h) were not significantly different for bees fed Hv1a/GNA prior to conditioning; con, control; Hv1a, Hv1a/GNA. Data represent mean response probabilities ± 95% CIs. (Online version in colour.)

Experiments based on an olfactory conditioning protocol were performed to assess whether Hv1a/GNA affected olfactory learning and memory in the honeybee following both acute and long-term oral exposure (figure 2). Studies to investigate potential effects of acute exposure also included a positive control for testing the effects of a CaV channel blocker on this behavioural parameter (benidipine hydrochloride; Ben), since a CaV channel is the target of the Hv1a toxin. As shown in figure 2*a*, there was an overall difference in the rate of learning between the different acute treatment groups (lreg, 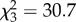, *p* < 0.001). Ben (positive control) impaired the rate of olfactory learning by up to 50% over the course of six conditioning trials (lsc, *p* = 0.026). The rate of learning was unaffected when bees were treated with an acute dose of either Hv1a/GNA (lsc, *p* = 0.957) or GNA (lsc, *p* = 0.702) (figure 2*a*). Treatment influenced the expression of short-term memory (STM, figure 2*b*); bees fed Ben had lower responses than the control, GNA- or the Hv1a/GNA-treated bees (lreg, STM, 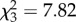, *p* = 0.050; lsc for the control versus Ben, *p* = 0.025). However, when tested for long-term memory (LTM) 24 h later, there was no significant difference in the rate of response to the conditioned odour between the treatment groups (lreg, LTM, 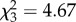, *p* = 0.197). For both tests, the rate of response was always greater towards the conditioned odour than a novel odour (data not shown, lreg, STM, 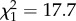, *p* < 0.001; LTM, 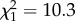, *p* = 0.001).

The effects of chronic oral exposure to Hv1a/GNA on olfactory learning ability and memory were also tested. The results showed that Hv1a/GNA did not influence the rate or asymptotic level of learning when compared to the control (lreg, 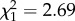, *p* = 0.107; figure 2*c*). Similarly, bees fed Hv1a/GNA did not exhibit impaired STM or LTM performance (lreg, STM, 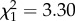, *p* = 0.069; LTM, 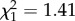, *p* = 0.235; figure 2*d*). These results demonstrate that the fusion protein HV1a/GNA does not impair olfactory learning or memory formation, even though a positive control for the same target as the fusion protein (Ben) significantly reduced the rate of learning and STM.

### (c) Detection of Hv1a/GNA in honeybee tissues by western blotting

**Figure 3.**
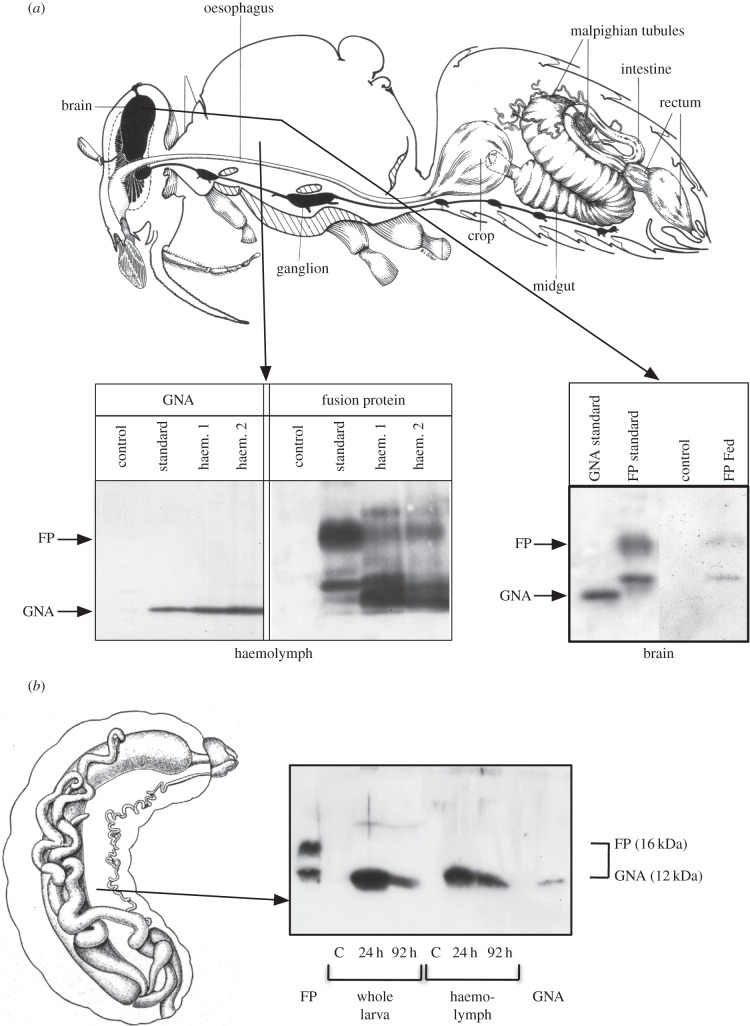
Immuno-assay by western blotting demonstrates internalization of Hv1a/GNA in adult honeybee tissues. Bands of GNA (12 kDa) and Hv1a/GNA (FP; 16 kDa) are indicated. (*a*) Diagram of adult honeybee showing the presence of GNA and fusion protein Hv1a/GNA (FP) in both the haemolymph and brain after feeding solutions containing proteins. Insects were fed 100 µg GNA or Hv1a/GNA, and haemolymph or brain tissue was collected after 24 h for analysis. (*b*) Diagram of larval honeybee showing that Hv1a/GNA (FP) is degraded after ingestion; larvae were dosed with 100 µg Hv1a/GNA per larva and haemolymph was collected after 24 h for analysis.

To investigate potential internalization of HV1a/GNA in both adult and larval honeybees, tissue samples were collected from insects fed on diet containing either GNA or Hv1a/GNA 24 h after exposure and subsequently transferred to diet without treatment for varying times. In adult bees, the Hv1a/GNA fusion protein was clearly visualized in haemolymph samples 24 h after feeding (figure 3*a*), demonstrating that the GNA carrier component was able to direct transport of the toxin component across the gut epithelium, as has been observed in other insects [21]. Fusion protein was also detectable in brain tissue, showing that the toxin had been able to reach its site of action in the CNS, and that the lack of toxicity of Hv1a/GNA was not owing to failure to transport or access its target. As in adult bees, the western blotting experiment for bee larvae showed evidence for transport of the GNA carrier across the gut epithelium, since GNA was present both in haemolymph and whole insect after feeding and chase (24 and 92 h). However, no evidence for toxin transport was seen, as all the fusion protein was degraded and no intact Hv1a/GNA could be detected (figure 3*b*). As expected, the levels of degraded protein, representing the GNA part of the fusion protein, were reduced by the longer chase period of 92 h compared with 24 h. The absence of toxicity of Hv1a/GNA to larval bees is thus primarily owing to protein degradation in the gut preventing transport of the toxin to its sites of action, although on the basis of results from adult bees, it is likely that the toxin would not affect calcium channels if transported to the haemolymph.

## 4. Discussion

The fusion protein Hv1a/GNA complies with the current European and American risk assessments for pesticide toxicity to honeybees, as tests described in the OECD guidelines were fulfilled [23,24]. Following those assays, acute oral and contact toxicity of Hv1a/GNA can be considered negligible (LD_50_ > 100 µg bee^−1^). Even when bees were injected with Hv1a/GNA, only 17% of the bees died within 48 h. In comparison, lepidopteran larvae injected with comparable amounts of fusion protein typically show a 90–100% reduction in survival [21]. We assume this level of mortality in bees can be considered low, as, according to the United States Environmental Protection Agency, compounds with *contact* toxicity of LD_50_ > 11 µg bee^−1^ are classified as ‘relatively non-toxic’ [38]. This suggests that the omega toxin does not reach or bind to the target site of action in the CNS of bees as avidly as it does in lepidopteran larvae, or that there are critical differences in the ion channel binding sites in bees and lepidopteran larvae. Surprisingly, the survival of bees injected with GNA was significantly reduced (*ca* 60%), when compared with the control treatment, whereas the injection of equivalent, high doses of GNA into lepidopteran larvae does not result in substantial mortality. In our experiments, GNA was only used as a control, in the event that the fusion protein had an influence on survival, learning and memory. Previous results of feeding bioassays have suggested that plant lectins have differing effects on insects, although the basis of this effect remains unclear. Hv1a/GNA did not have a measurable influence on survival or cognition in adult worker honeybees after acute or long-term oral exposure. The observed lack of Hv1a/GNA toxicity contrasts with lethal effects of neonicotinoids used as positive controls: Ace was acutely toxic at similar concentrations to those previously reported [34], and chronic TMX ingestion at a field-relevant dose had significant lethal effects at the concentrations found in nectar and pollen [36,37].

No adverse effects of Hv1a/GNA on honeybee learning and memory were detected in the assays reported here, in spite of the fact that the doses we gave the bees prior to the assay were relatively high. In fact, the chronic exposure experiment is likely to have provided a dose to the bees far above what they would experience in the field; this is because the biopesticide is applied as a spray and not as a systemic pesticide and so would not be consumed in large amounts by bees in nectar and pollen. Previous studies have found that exposure to field-relevant doses of pesticides which target the CNS, such as neonicotinoids and organophosphates, impair the ability of honeybees to learn and remember the association between an olfactory cue and a sucrose reward [7,8]. The effect of Hv1a on insect calcium channels [20] suggests that it could have significant effects on learning and memory, especially if CaV channels are affected [39]. CaV channels are known to play a role in olfactory learning in mammals [40] and are present in the areas of the honeybee brain, where olfactory associations are processed [41,42]. This prediction of CaV involvement in honeybee learning was confirmed, as the positive control for CaV block, benidipine HCl [43], impaired olfactory learning and STM. What was surprising, however, was that benidipine HCl (used as a positive control) did not influence long-term olfactory memory. A previous study of the influence of calcium on olfactory learning and memory in bees showed that blocking intracellular calcium release prior to conditioning impaired LTM formation [39]. Instead of blocking CaV channels as we did, however, this study used a chelator of calcium to prevent calcium binding to CaV channels. By contrast, Hv1a/GNA had no significant effect on olfactory learning or memory, indicating that at the doses we tested, it is an ineffective antagonist of the CaV channel in the honeybee brain.

This lack of observed adverse effects on either the survival or the learning ability of adult honeybees was not owing to the fusion protein failing to reach the target site in the CNS. When orally administered to adult worker honeybees, Hv1a/GNA was capable of crossing the epithelial gut wall, as Hva1/GNA immunoreactivity was detected in the haemolymph and brain tissue 1 h after ingestion. In contrast with adult honeybees, larvae were capable of cleaving the fusion protein within the digestive tract, preventing Hv1a/GNA from reaching the site of action. A decline in gut proteolytic activity is known to occur as bees develop into foragers [44,45], reflecting the high protein content of the diet consumed by larval bees, in contrast to the low-protein nectar diet consumed by adults.

It would appear that despite reaching the CNS of adult bees, Hv1a/GNA does not block the CaV channels of *Apis mellifera*. Conversely, another peptide isolated from *H. versuta* venom, ω-ACTX-Hv2a, has been shown to block CaV channels in honeybee brain neurons [46]. Although this protein has a similar disulfide connection pattern to Hv1a, it has only limited sequence similarity, which could account for differences in toxicity towards bees. Hv1a has insecticidal activity against Lepidoptera such as *Helicoverpa armigera* [47] and has been shown to block CaV currents in CNS neurons from *Drosophila melanogaster*, and the cockroach *Periplaneta americana* [19,20]. However, compared with other insecticide targets in the CNS such as acetylcholine receptors and NaV channels, CaV channels are less well conserved between different insect orders [48], thus conferring a certain degree of specificity. Functional expression of recombinant CaV channels from different insect orders would be necessary to fully elucidate the basis of this differential sensitivity to Hv1a.

The data we report here suggest that Hv1a/GNA is a potentially specific biopesticide, as it shows no adverse effects on the honeybee, *Apis mellifera*, an economically important pollinator, while being toxic to agronomically important insect pests. Another possible reason for this lack of toxicity towards honeybee is owing to its degradation within the bee, preventing accumulation of the fusion protein even if exposure is repeated. The experiments we have performed exceed current European and American requirements for pesticide safety, and include an olfactory learning assay, which found no adverse effects of Hv1a/GNA on this behavioural parameter. These results show that Hv1a/GNA can be considered safer for honeybees than some currently used pesticides, such as neonicotinoids, although additional safety tests should be performed to confirm its safety against other beneficial hymenoptera, such as bumblebees and parasitoid wasps. This study also highlights the need to extend current guidelines for the safety testing of new pesticides to include behavioural studies, particularly for pollinating insects.

## Supplementary Material

ESM Figure 1

## Supplementary Material

data bees
