# Supplementary material for: Novel biopesticide based on a spider venom peptide shows no adverse effects on honeybees
Source: Proc Biol Sci. 2014 Jul 22;281(1787):20140619. doi: 10.1098/rspb.2014.0619 (PMC4071547; doi:10.1098/rspb.2014.0619)
Supplement: ESM Figure 1 [file rspb20140619supp1.pdf]

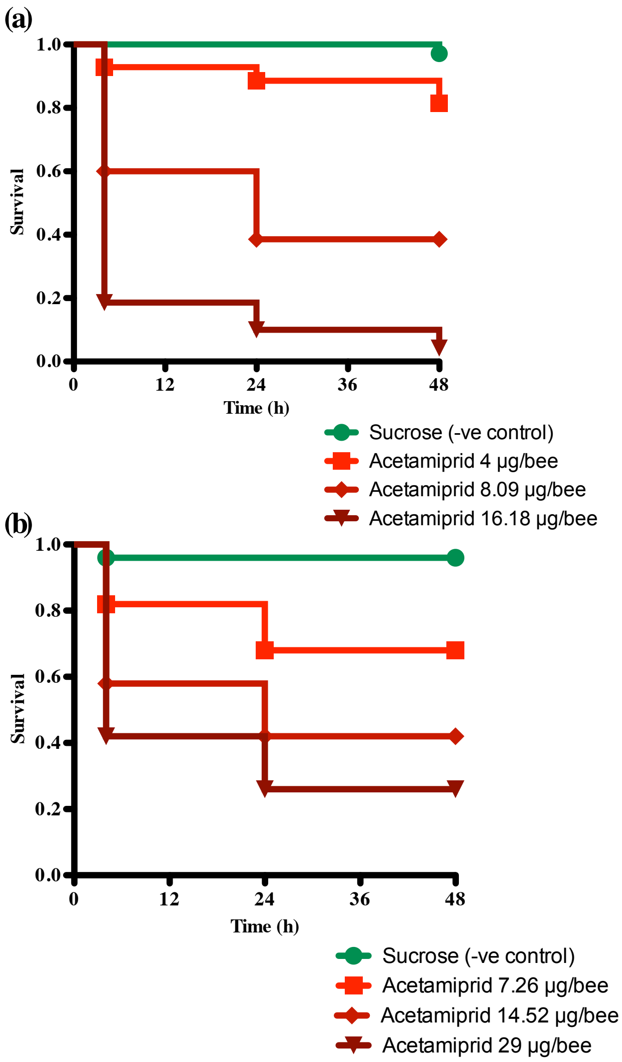

ESM Figure 1:

Dose response for the insecticide acetamiprid for a) acute contact toxicity (4, 8.09 and 16.18 µg/bee) and, b) acute oral toxicity (7.26, 14.52 and 29 µg/bee). The intermediate dose tested for both assays represents the published LD50 value for the pesticides for bees (EU, SANCO/1392/2001). These LD50 valuses were confirmed in the present study.
