# Supplementary material for: Novel biopesticide based on a spider venom peptide shows no adverse effects on honeybees
Source: Proc Biol Sci. 2014 Jul 22;281(1787):20140619. doi: 10.1098/rspb.2014.0619 (PMC4071547; doi:10.1098/rspb.2014.0619)
Supplement: data bees [file rspb20140619supp2.pdf]

learning chronic 350ppm restructured

7 day feeding expt

trt 1 = sucrose only control
2 = 350 ppm Hv1a/GNA

trialno = training trials of learning expt, 1-6

response = did bee perform proboscis extension?
1 = yes
0 = no

id	trt	trialno	response
1	1	1	0
1	1	2	1
1	1	3	1
1	1	4	1
1	1	5	1
1	1	6	1
2	1	1	0
2	1	2	0
2	1	3	0
2	1	4	0
2	1	5	1
2	1	6	1
3	1	1	0
3	1	2	1
3	1	3	1
3	1	4	1
3	1	5	1
3	1	6	1
4	1	1	0
4	1	2	1
4	1	3	1
4	1	4	1
4	1	5	1
4	1	6	1
5	1	1	0
5	1	2	0
5	1	3	1
5	1	4	1
5	1	5	1
5	1	6	1
6	1	1	0
6	1	2	1
6	1	3	1
6	1	4	1
6	1	5	1
6	1	6	1
7	1	1	0
7	1	2	0
7	1	3	1
7	1	4	1
7	1	5	0
7	1	6	1
8	1	1	0
8	1	2	1
8	1	3	1
8	1	4	1
8	1	5	1
8	1	6	1
9	1	1	0
9	1	2	0
9	1	3	1
9	1	4	0
9	1	5	0
9	1	6	0
10	1	1	0
10	1	2	0
10	1	3	0
10	1	4	1
10	1	5	1
10	1	6	0
11	2	1	0
11	2	2	0
11	2	3	0

11	2	4	1
11	2	5	1
11	2	6	1
12	2	1	0
12	2	2	1
12	2	3	0
12	2	4	0
12	2	5	1
12	2	6	1
13	2	1	0
13	2	2	1
13	2	3	1
13	2	4	1
13	2	5	1
13	2	6	1
14	2	1	0
14	2	2	0
14	2	3	1
14	2	4	1
14	2	5	1
14	2	6	1
15	2	1	0
15	2	2	1
15	2	3	1
15	2	4	1
15	2	5	1
15	2	6	1
16	2	1	0
16	2	2	1
16	2	3	1
16	2	4	1
16	2	5	1
16	2	6	1
17	2	1	0
17	2	2	1
17	2	3	1
17	2	4	1
17	2	5	1
17	2	6	1
18	2	1	0
18	2	2	1
18	2	3	1
18	2	4	1
18	2	5	0
18	2	6	1
19	1	1	0
19	1	2	1
19	1	3	1
19	1	4	0
19	1	5	1
19	1	6	1
20	1	1	0
20	1	2	0
20	1	3	0
20	1	4	1
20	1	5	1
20	1	6	0
21	1	1	0
21	1	2	0
21	1	3	1
21	1	4	1
21	1	5	1
21	1	6	1
22	1	1	0

22	1	2	1
22	1	3	0
22	1	4	0
22	1	5	0
22	1	6	0
23	1	1	0
23	1	2	0
23	1	3	0
23	1	4	0
23	1	5	0
23	1	6	1
24	1	1	0
24	1	2	1
24	1	3	1
24	1	4	1
24	1	5	0
24	1	6	0
25	1	1	0
25	1	2	0
25	1	3	1
25	1	4	1
25	1	5	1
25	1	6	1
26	1	1	0
26	1	2	0
26	1	3	1
26	1	4	1
26	1	5	1
26	1	6	1
27	1	1	0
27	1	2	1
27	1	3	1
27	1	4	0
27	1	5	1
27	1	6	1
28	2	1	0
28	2	2	0
28	2	3	1
28	2	4	0
28	2	5	1
28	2	6	1
29	2	1	0
29	2	2	1
29	2	3	0
29	2	4	1
29	2	5	0
29	2	6	0
30	2	1	0
30	2	2	0
30	2	3	1
30	2	4	1
30	2	5	1
30	2	6	1
31	2	1	0
31	2	2	1
31	2	3	1
31	2	4	1
31	2	5	1
31	2	6	1
32	2	1	0
32	2	2	0
32	2	3	0
32	2	4	1
32	2	5	0

32	2	6	0
33	2	1	0
33	2	2	1
33	2	3	1
33	2	4	0
33	2	5	1
33	2	6	1
34	1	1	0
34	1	2	0
34	1	3	0
34	1	4	0
34	1	5	1
34	1	6	1
35	1	1	0
35	1	2	0
35	1	3	0
35	1	4	0
35	1	5	1
35	1	6	1
36	1	1	0
36	1	2	1
36	1	3	1
36	1	4	1
36	1	5	1
36	1	6	1
37	1	1	0
37	1	2	0
37	1	3	0
37	1	4	1
37	1	5	1
37	1	6	1
38	1	1	0
38	1	2	1
38	1	3	1
38	1	4	1
38	1	5	1
38	1	6	1
39	1	1	0
39	1	2	0
39	1	3	0
39	1	4	1
39	1	5	0
39	1	6	0
40	1	1	0
40	1	2	0
40	1	3	1
40	1	4	1
40	1	5	1
40	1	6	1
41	2	1	0
41	2	2	0
41	2	3	0
41	2	4	1
41	2	5	1
41	2	6	1
42	2	1	0
42	2	2	0
42	2	3	0
42	2	4	0
42	2	5	1
42	2	6	1
43	2	1	0
43	2	2	0
43	2	3	1

43	2	4	1
43	2	5	1
43	2	6	1
44	2	1	0
44	2	2	1
44	2	3	1
44	2	4	1
44	2	5	1
44	2	6	1
45	2	1	0
45	2	2	0
45	2	3	1
45	2	4	1
45	2	5	1
45	2	6	1
46	2	1	0
46	2	2	0
46	2	3	1
46	2	4	1
46	2	5	1
46	2	6	1

trt	resp	time	odour	**memory chronic 350ppm restructured**	
1	1	2	1		
1	1	2	1		7 day feeding expt
1	1	2	1	trt	1 = sucrose fed control
1	0	2	1		2 = 350 ppm Hv1a/GNA
1	1	2	1		
1	1	2	1		
1	1	2	1		odour
1	1	2	1		1 = hexanol (trained odour)
1	0	2	1		2 = octanol (novel odour)
1	0	2	1		
2	1	2	1		
2	0	2	1	timepoint	
2	1	2	1	1 = 6th learning trial	
2	1	2	1	2 = 10 mins later (short term memory)	
2	1	2	1	3 = 24h later (long term memory)	
2	1	2	1		
2	1	2	1		
2	0	2	1	response = did bee perform proboscis extension?	
1	1	2	1	1 = yes	
1	0	2	1	0 = no	
1	1	2	1		
1	0	2	1		
1	1	2	1		
1	1	2	1		
1	1	2	1		
1	1	2	1		
1	0	2	1		
2	1	2	1		
2	0	2	1		
2	1	2	1		
2	1	2	1		
2	1	2	1		
2	1	2	1		
1	0	2	1		
1	1	2	1		
1	1	2	1		
1	0	2	1		
1	0	2	1		

1	1	2	1
1	0	2	1
2	1	2	1
2	1	2	1
2	0	2	1
2	1	2	1
2	1	2	1
2	0	2	1
1	0	2	2
1	0	2	2
1	0	2	2
1	0	2	2
1	0	2	2
1	0	2	2
1	0	2	2
1	1	2	2
1	0	2	2
1	0	2	2
2	0	2	2
2	0	2	2
2	0	2	2
2	0	2	2
2	1	2	2
2	0	2	2
2	0	2	2
2	0	2	2
1	0	2	2
1	0	2	2
1	1	2	2
1	0	2	2
1	0	2	2
1	1	2	2
1	1	2	2
1	0	2	2
1	0	2	2
2	1	2	2
2	0	2	2
2	0	2	2
2	0	2	2
2	0	2	2
2	1	2	2
1	0	2	2
1	0	2	2
1	0	2	2
1	0	2	2
1	0	2	2
1	0	2	2
1	0	2	2
2	0	2	2
2	0	2	2
2	1	2	2
2	0	2	2
2	0	2	2
2	0	2	2
1	1	3	1
1	0	3	1
1	1	3	1
1	0	3	1
1	1	3	1
1	1	3	1
1	1	3	1
1		3	1
1	0	3	1
1		3	1

2	1	3	1
2		3	1
2	1	3	1
2	1	3	1
2	1	3	1
2	1	3	1
2	0	3	1
2	0	3	1
1	1	3	1
1	1	3	1
1	1	3	1
1	0	3	1
1	0	3	1
1	0	3	1
1	1	3	1
1	0	3	1
1	0	3	1
2	1	3	1
2	0	3	1
2	1	3	1
2	0	3	1
2	0	3	1
2	1	3	1
1	0	3	1
1	0	3	1
1	1	3	1
1	0	3	1
1	1	3	1
1	0	3	1
1	1	3	1
2	0	3	1
2	0	3	1
2		3	1
2	1	3	1
2	1	3	1
2	1	3	1
1	0	3	2
1	0	3	2
1	0	3	2
1	0	3	2
1	1	3	2
1	1	3	2
1	1	3	2
1		3	2
1	0	3	2
1		3	2
2	0	3	2
2		3	2
2	1	3	2
2	1	3	2
2	0	3	2
2	1	3	2
2	0	3	2
2	0	3	2
1	1	3	2
1	1	3	2
1	1	3	2
1	0	3	2
1	0	3	2
1	0	3	2
1	1	3	2
1	0	3	2
1	0	3	2
2	0	3	2

2	0	3	2
2	0	3	2
2	0	3	2
2	0	3	2
2	0	3	2
1	0	3	2
1	0	3	2
1	0	3	2
1	0	3	2
1	0	3	2
1	0	3	2
1	0	3	2
2	0	3	2
2	0	3	2
2		3	2
2	0	3	2
2	0	3	2
2	1	3	2
1	1	1	1
1	1	1	1
1	1	1	1
1	1	1	1
1	1	1	1
1	1	1	1
1	1	1	1
1	1	1	1
1	0	1	1
1	0	1	1
2	1	1	1
2	1	1	1
2	1	1	1
2	1	1	1
2	1	1	1
2	1	1	1
2	1	1	1
2	1	1	1
1	1	1	1
1	0	1	1
1	1	1	1
1	0	1	1
1	1	1	1
1	0	1	1
1	1	1	1
1	1	1	1
1	1	1	1
2	1	1	1
2	1	1	1
2	1	1	1
2	0	1	1
2	1	1	1
2	1	1	1
1	1	1	1
1	1	1	1
1	1	1	1
1	1	1	1
1	0	1	1
1	1	1	1
1	1	1	1
2	1	1	1
2	1	1	1
2	1	1	1
2	1	1	1
2	1	1	1
2	1	1	1

100ug acute data

trt (treatment)

1 = sucrose fed control
2 = Hv1a/GNA
3 = benidipine HCl

4 = GNA

training trials of learning expt, T1-6

binary data shows did bee perform proboscis extension in response to odour?

1 = yes
0 = no

trt	T1	T2	T3	T4	T5	T6
1	0	1	1	0	1	1
1	0	0	1	1	1	1
1	0	0	1	1	1	1
1	0	1	0	0	0	1
1	0	0	1	0	1	0
1	0	1	1	1	1	1
1	0	1	1	1	1	1
1	0	0	1	1	1	1
1	0	1	1	1	1	1
1	0	0	0	1	1	0
1	0	0	1	1	0	1
2	0	1	1	1	1	1
2	0	0	0	1	1	1
2	0	0	1	1	1	1
2	0	0	0	0	0	1
2	0	0	0	0	0	0
2	0	0	1	0	1	0
2	0	1	1	1	1	0
2	0	0	0	0	0	1
2	0	0	0	0	0	0
2	0	0	1	1	1	1
2	0	1	1	1	1	1
3	0	0	1	1	0	1
3	0	1	1	1	1	1
3	0	1	0	0	1	0
3	0	0	1	0	1	1
3	0	0	0	0	0	0
3	0	0	0	0	0	0
3	0	1	1	0	0	1
3	0	0	0	0	0	1
3	0	0	0	1	0	0
3	0	0	0	0	1	0
3	0	1	0	1	0	1
3	0	0	0	1	0	0
3	0	0	0	0	0	0
4	0	1	1	1	1	0
4	0	1	1	1	1	1
4	0	0	0	0	0	0
4	0	0	1	1	1	1
4	0	0	1	1	0	1
4	0	1	0	0	0	0
4	0	0	0	1	1	1
4	0	0	1	1	1	1
4	0	0	0	0	1	1
4	0	1	1	1	1	1
4	0	1	1	1	1	1
4	0	1	1	1	0	1

4	0	0	0	0	1	1
1	0	0	1	1	1	1
1	0	1	1	1	1	1
1	0	1	1	1	1	1
1	0	0	1	1	1	1
1	0	1	1	1	1	1
1	0	0	1	0	0	0
1	0	0	0	0	1	1
1	0	0	0	0	0	0
2	0	0	1	1	1	1
2	0	0	0	0	1	1
2	0	1	1	1	1	1
2	0	0	1	1	1	1
2	0	0	0	0	0	0
2	0	1	1	1	1	1
2	0	1	1	1	1	1
2	0	0	1	1	1	1
4	0	0	0	0	0	0
4	0	0	1	1	1	1
4	0	0	1	1	1	1
4	0	1	1	1	1	1
4	0	1	1	1	1	1
4	0	1	1	1	1	1
4	0	1	1	1	1	1
4	0	0	1	0	0	0
4	0	0	1	1	1	1
4	0	0	1	1	1	1
3	0	0	0	0	1	1
3	0	0	0	0	0	0
3	0	0	1	1	1	1
3	0	0	1	0	1	0
3	0	0	0	0	0	0
3	0	0	0	0	0	1
3	0	1	1	1	1	1
3	0	0	1	1	1	0
3	0	0	1	0	1	1
3	0	0	0	0	0	0

trt	Response	timepoint	odour	**100ug acute memory**
1	1	1	1	trt (treatment)
1	1	1	1	1 = sucrose fed control
1	1	1	1	2 = Hv1a/GNA
1	1	1	1	3 = benidipine HCl
1	0	1	1	4 = GNA
1	1	1	1	
1	1	1	1	odour
1	1	1	1	1 = hexanol (trained odour)
1	1	1	1	2 = octanol (novel odour)
1	0	1	1	
1	1	1	1	
2	1	1	1	timepoint
2	1	1	1	1 = 6th learning trial
2	1	1	1	2 = 10 mins later (short term memory)
2	1	1	1	3 = 24h later (long term memory)
2	0	1	1	
2	0	1	1	
2	0	1	1	response = did bee perform proboscis extension?
2	1	1	1	1 = yes
2	0	1	1	0 = no
2	1	1	1	
2	1	1	1	
3	1	1	1	

3	1	1	1
3	0	1	1
3	1	1	1
3	0	1	1
3	0	1	1
3	1	1	1
3	1	1	1
3	0	1	1
3	0	1	1
3	1	1	1
3	0	1	1
3	0	1	1
4	0	1	1
4	1	1	1
4	0	1	1
4	1	1	1
4	1	1	1
4	0	1	1
4	1	1	1
4	1	1	1
4	1	1	1
4	1	1	1
4	1	1	1
4	1	1	1
4	1	1	1
1	1	1	1
1	1	1	1
1	1	1	1
1	1	1	1
1	1	1	1
1	0	1	1
1	1	1	1
1	0	1	1
2	1	1	1
2	1	1	1
2	1	1	1
2	1	1	1
2	0	1	1
2	1	1	1
2	1	1	1
2	1	1	1
4	0	1	1
4	1	1	1
4	1	1	1
4	1	1	1
4	1	1	1
4	1	1	1
4	1	1	1
4	0	1	1
4	1	1	1
4	1	1	1
3	1	1	1
3	0	1	1
3	1	1	1
3	0	1	1
3	0	1	1
3	1	1	1
3	1	1	1
3	0	1	1
3	1	1	1
3	0	1	1
1	0	2	1
1	1	2	1
1	1	2	1

1	0	2	1
1	0	2	1
1	1	2	1
1	1	2	1
1	1	2	1
1	1	2	1
1	0	2	1
1	0	2	1
2	1	2	1
2	1	2	1
2	1	2	1
2	0	2	1
2	0	2	1
2	0	2	1
2	0	2	1
2	0	2	1
2	0	2	1
2	1	2	1
2	1	2	1
3	0	2	1
3	1	2	1
3	0	2	1
3	1	2	1
3	0	2	1
3	1	2	1
3	0	2	1
3	0	2	1
3	0	2	1
3	0	2	1
3	0	2	1
3	0	2	1
3	0	2	1
4	0	2	1
4	1	2	1
4	0	2	1
4	1	2	1
4	1	2	1
4	0	2	1
4	1	2	1
4	0	2	1
4	1	2	1
4	1	2	1
4	0	2	1
4	1	2	1
4	1	2	1
1	1	2	1
1	1	2	1
1	1	2	1
1	1	2	1
1	1	2	1
1	1	2	1
1	0	2	1
1	0	2	1
2	1	2	1
2	1	2	1
2	0	2	1
2	1	2	1
2	0	2	1
2	1	2	1
2	1	2	1
2	1	2	1
4	0	2	1
4	1	2	1
4	1	2	1

4	1	2	1
4	0	2	1
4	0	2	1
4	1	2	1
4	0	2	1
4	0	2	1
4	1	2	1
3	1	2	1
3	0	2	1
3	1	2	1
3	0	2	1
3	0	2	1
3	0	2	1
3	1	2	1
3	0	2	1
3	1	2	1
3	0	2	1
1	0	2	2
1	0	2	2
1	0	2	2
1	0	2	2
1	0	2	2
1	0	2	2
1	0	2	2
1	0	2	2
1	0	2	2
1	0	2	2
1	0	2	2
2	0	2	2
2	0	2	2
2	1	2	2
2	0	2	2
2	0	2	2
2	0	2	2
2	0	2	2
2	0	2	2
2	0	2	2
2	0	2	2
2	1	2	2
3	0	2	2
3	0	2	2
3	0	2	2
3	0	2	2
3	0	2	2
3	0	2	2
3	0	2	2
3	0	2	2
3	0	2	2
3	0	2	2
3	0	2	2
3	0	2	2
3	0	2	2
4	0	2	2
4	1	2	2
4	0	2	2
4	1	2	2
4	1	2	2
4	0	2	2
4	1	2	2
4	0	2	2
4	1	2	2
4	1	2	2
4	0	2	2
4	0	2	2

4	1	2	2
1	1	2	2
1	1	2	2
1	0	2	2
1	0	2	2
1	1	2	2
1	0	2	2
1	0	2	2
1	0	2	2
2	0	2	2
2	0	2	2
2	0	2	2
2	1	2	2
2	0	2	2
2	0	2	2
2	1	2	2
2	0	2	2
4	0	2	2
4	0	2	2
4	0	2	2
4	0	2	2
4	0	2	2
4	0	2	2
4	1	2	2
4	0	2	2
4	0	2	2
4	0	2	2
3	0	2	2
3	0	2	2
3	0	2	2
3	0	2	2
3	0	2	2
3	0	2	2
3	1	2	2
3	0	2	2
3	0	2	2
3	0	2	2
1	1	3	1
1	0	3	1
1	0	3	1
1	1	3	1
1	1	3	1
1	1	3	1
1	1	3	1
1	0	3	1
1	1	3	1
1	1	3	1
1		3	1
2	1	3	1
2	1	3	1
2	0	3	1
2	0	3	1
2	0	3	1
2	0	3	1
2	0	3	1
2	0	3	1
2	1	3	1
2	0	3	1
2	1	3	1
3	1	3	1
3	0	3	1
3	1	3	1
3	1	3	1
3	0	3	1

3	0	3	1
3	0	3	1
3		3	1
3		3	1
3		3	1
3		3	1
3		3	1
3		3	1
4	0	3	1
4	1	3	1
4	0	3	1
4	1	3	1
4	1	3	1
4	0	3	1
4	1	3	1
4	0	3	1
4	1	3	1
4	1	3	1
4	0	3	1
4	1	3	1
4	1	3	1
1	1	3	1
1	0	3	1
1	1	3	1
1	0	3	1
1	0	3	1
1	1	3	1
1		3	1
1		3	1
2	0	3	1
2	1	3	1
2	0	3	1
2	0	3	1
2	0	3	1
2	1	3	1
2	1	3	1
2		3	1
4	0	3	1
4	1	3	1
4	1	3	1
4	1	3	1
4	1	3	1
4	0	3	1
4	0	3	1
4	1	3	1
4		3	1
4		3	1
3	1	3	1
3	1	3	1
3	1	3	1
3	1	3	1
3	0	3	1
3	0	3	1
3	1	3	1
3	0	3	1
3	1	3	1
3	0	3	1
1	0	3	2
1	0	3	2
1	0	3	2
1	0	3	2
1	0	3	2
1	1	3	2
1	1	3	2

1	0	3	2
1	1	3	2
1	1	3	2
1		3	2
2	0	3	2
2	0	3	2
2	0	3	2
2	0	3	2
2	0	3	2
2	0	3	2
2	0	3	2
2	1	3	2
2	0	3	2
2	0	3	2
2	0	3	2
3	0	3	2
3	0	3	2
3	0	3	2
3	0	3	2
3	0	3	2
3	0	3	2
3	0	3	2
3		3	2
3		3	2
3		3	2
3		3	2
3		3	2
3		3	2
4	0	3	2
4	1	3	2
4	0	3	2
4	1	3	2
4	0	3	2
4	0	3	2
4	1	3	2
4	0	3	2
4	1	3	2
4	1	3	2
4	1	3	2
4	1	3	2
4	0	3	2
1	0	3	2
1	0	3	2
1	0	3	2
1	0	3	2
1	0	3	2
1	1	3	2
1		3	2
1		3	2
2	1	3	2
2	0	3	2
2	0	3	2
2	0	3	2
2	0	3	2
2	1	3	2
2	0	3	2
2		3	2
4	0	3	2
4	0	3	2
4	0	3	2
4	1	3	2
4	0	3	2
4	0	3	2
4	0	3	2

4	0	3	2
4		3	2
4		3	2
3	0	3	2
3	0	3	2
3	1	3	2
3	1	3	2
3	0	3	2
3	1	3	2
3	1	3	2
3	0	3	2
3	0	3	2
3	0	3	2

100ug acute data learning and memory averages

	sucrose	GNA	Benidipine	Hv1a/GNA		hex 10	octan24
1	0	0	0	0	con	0.6667	0.1667
2	0.4444	0.375	0.2941	0.4762	GNA	0.6875	0.25
3	0.8333	0.75	0.5294	0.8095	ben	0.3529	0.0588
4	0.7222	0.75	0.4706	0.8095	Hv1a	0.619	0.381
5	0.8333	0.875	0.5882	0.8095		hex 24	octan24
6	0.8333	0.875	0.6471	0.8571	con	0.625	0.3125
					GNA	0.4	0.2
					benid	0.6667	0.3333
					FP5	0.6842	0.4211

chronic assay learning and memory 350 ppm averages

trial	control	hv1a/GNA				hex10	octan10
1	0	0			STM con	0.6154	0.1538
2	0.4231	0.5			STM Hv1a	0.75	0.2
3	0.6538	0.7			LTM con	0.5	0.2917
4	0.6923	0.8			LTM Hv1a	0.6111	0.2222
5	0.7692	0.85				hex24	octan24
6	0.7692	0.9			LTM control	0.5	0.2917
					LTM Hv1a	0.6111	0.2222

For all assays below: 1= event; 0=censor

**Contact**	ctrl time	ctrl status	GNA time	GNA status
	48	1	48	1
	48	1	48	1
	48	0	48	1
	48	0	48	1
	48	0	48	1
	48	0	48	0
	48	0	48	0
	48	0	48	0
	48	0	48	0
	48	0	48	0
	48	0	48	0
	48	0	48	0
	48	0	48	0
	48	0	48	0

48	0	48	0
48	0	48	0
48	0	48	0
48	0	48	0
48	0	48	0
48	0	48	0
48	0	48	0
48	0	48	0
48	0	48	0
48	0	48	0
48	0	48	0
48	0	48	0
48	0	48	0
48	0	48	0
48	0	48	0
48	0	48	0
48	0	48	0
48	0	48	0
48	0	48	0
48	0	48	0
48	0	48	0
48	0	48	0
48	0	48	0
48	0	48	0
48	0	48	0
48	0	48	0
48	0	48	0
48	0	48	0
48	0	48	0
48	0	48	0
48	0	48	0
48	0	48	0
48	0	48	0
48	0	48	0
48	0	48	0
48	0	48	0
48	0	48	0
48	0	48	0
48	0	48	0
48	0	48	0
48	0	48	0
48	0	48	0
48	0	48	0
48	0	48	0
48	0	48	0
48	0	48	0
48	0	48	0
48	0	48	0
48	0	48	0
48	0	48	0
48	0	48	0
48	0	48	0
48	0	48	0
48	0	48	0
48	0	48	0
48	0	48	0

Hv1a/GNA tim	Hv1a/GNA stat	Acet time	Acet status
48	1	4	1
48	1	4	1
48	1	4	1
48	1	4	1
48	1	4	1
48	0	4	1

48	0	4	1
48	0	4	1
48	0	4	1
48	0	4	1
48	0	4	1
48	0	4	1
48	0	4	1
48	0	4	1
48	0	4	1
48	0	4	1
48	0	4	1
48	0	4	1
48	0	4	1
48	0	4	1
48	0	4	1
48	0	4	1
48	0	4	1
48	0	4	1
48	0	4	1
48	0	4	1
48	0	4	1
48	0	4	1
48	0	24	1
48	0	24	1
48	0	24	1
48	0	24	1
48	0	24	1
48	0	24	1
48	0	24	1
48	0	24	1
48	0	24	1
48	0	24	1
48	0	24	1
48	0	24	1
48	0	24	1
48	0	24	1
48	0	24	1
48	0	48	0
48	0	48	0
48	0	48	0
48	0	48	0
48	0	48	0
48	0	48	0
48	0	48	0
48	0	48	0
48	0	48	0
48	0	48	0
48	0	48	0
48	0	48	0
48	0	48	0
48	0	48	0
48	0	48	0
48	0	48	0
48	0	48	0
48	0	48	0
48	0	48	0
48	0	48	0
48	0	48	0
48	0	48	0
48	0	48	0
48	0	48	0
48	0	48	0
48	0	48	0
48	0	48	0

**Oral**	ctrl time	ctrl status	gna time	gna status
	4	1	24	1
	4	1	24	1
	48	0	48	1
	48	0	48	1
	48	0	48	1
	48	0	48	1
	48	0	48	1
	48	0	48	1
	48	0	48	1
	48	0	48	1
	48	0	48	1
	48	0	48	1
	48	0	48	1
	48	0	48	1
	48	0	48	1
	48	0	48	1
	48	0	48	1
	48	0	48	1
	48	0	48	1
	48	0	48	1
	48	0	48	0
	48	0	48	0
	48	0	48	0
	48	0	48	0
	48	0	48	0
	48	0	48	0
	48	0	48	0
	48	0	48	0
	48	0	48	0
	48	0	48	0
	48	0	48	0
	48	0	48	0
	48	0	48	0
	48	0	48	0
	48	0	48	0
	48	0	48	0
	48	0	48	0
	48	0	48	0
	48	0	48	0
	48	0	48	0
	48	0	48	0
	48	0	48	0
	48	0	48	0
	48	0	48	0
	48	0	48	0
	48	0	48	0
	48	0	48	0
	48	0	48	0
	48	0	48	0
	48	0	48	0
	48	0	48	0
	48	0	48	0
	48	0	48	0
	48	0	48	0
	48	0	48	0
	48	0	48	0
	48	0	48	0
	48	0	48	0
	48	0	48	0
	48	0	48	0

	Hv1a/GNA tim	Hv1a/GNA stat	acet 14 time	acet 14 status

4	1	4	1
4	1	4	1
4	1	4	1
4	1	4	1
48	1	4	1
48	1	4	1
48	1	4	1
48	1	4	1
48	1	4	1
48	1	4	1
48	1	4	1
48	1	4	1
48	0	4	1
48	0	4	1
48	0	4	1
48	0	4	1
48	0	4	1
48	0	4	1
48	0	4	1
48	0	4	1
48	0	4	1
48	0	24	1
48	0	24	1
48	0	24	1
48	0	24	1
48	0	24	1
48	0	24	1
48	0	24	1
48	0	24	1
48	0	48	0
48	0	48	0
48	0	48	0
48	0	48	0
48	0	48	0
48	0	48	0
48	0	48	0
48	0	48	0
48	0	48	0
48	0	48	0
48	0	48	0
48	0		
48	0		
48	0		
48	0		
48	0		
48	0		
48	0		
48	0		
48	0		
48	0		
48	0		
48	0		
48	0		
48	0		
48	0		
48	0		
48	0		
48	0		
48	0		
48	0		

**Injection**	ctrl time	ctrl status	gna time	gna status
	48	0	4	1
	48	0	24	1

48	0	24	1
48	0	24	1
48	0	24	1
48	0	24	1
48	0	24	1
48	0	24	1
48	0	24	1
48	0	48	1
48	0	48	1
48	0	48	1
48	0	48	1
48	0	48	1
48	0	48	1
48	0	48	1
48	0	48	1
48	0	48	0
48	0	48	0
48	0	48	0
48	0	48	0
48	0	48	0
48	0	48	0
48	0	48	0
48	0	48	0
48	0	48	0
48	0	48	0
48	0	48	0
48	0	48	0
48	0	48	0

Hv1a/GNA time	Hv1a/GNA status
24	1
48	1
48	1
48	1
48	1
48	0
48	0
48	0
48	0
48	0
48	0
48	0
48	0
48	0
48	0
48	0
48	0
48	0
48	0
48	0
48	0
48	0
48	0
48	0
48	0
48	0
48	0
48	0
48	0
48	0
